# HTLV-2 Encoded Antisense Protein APH-2 Suppresses HIV-1 Replication

**DOI:** 10.3390/v13081432

**Published:** 2021-07-23

**Authors:** Rajkumar Londhe, Smita Kulkarni

**Affiliations:** 1 Division of Virology, ICMR-National AIDS Research Institute, Pune 411026, Maharashtra, India; rajkumarlondhe@gmail.com; 2 Savitribai Phule Pune University, Pune 411007, Maharashtra, India

**Keywords:** APH-2, HTLV-2, HIV-1, replication, transcription, virus release

## Abstract

Antisense protein of Human T-cell Leukemia Virus Type 2 (HTLV-2), also called APH-2, negatively regulates the HTLV-2 and helps the virus to maintain latency via scheming the transcription. Despite the remarkable occurrence of HTLV-2/HIV-1 co-infection, the role of APH-2 influencing HIV-1 replication kinetics is poorly understood and needs investigation. In this study, we investigated the plausible role of APH-2 regulating HIV-1 replication. Herein, we report that the overexpression of APH-2 not only hampered the release of HIV-1 pNL4.3 from 293T cells in a dose-dependent manner but also affected the cellular gag expression. A similar and consistent effect of APH-2 overexpression was also observed in case of HIV-1 gag expression vector HXB2 pGag-EGFP. APH-2 overexpression also inhibited the ability of HIV-1 Tat to transactivate the HIV-1 LTR-driven expression of luciferase. Furthermore, the introduction of mutations in the IXXLL motif at the N-terminal domain of APH-2 reverted the inhibitory effect on HIV-1 Tat-mediated transcription, suggesting the possible role of this motif towards the downregulation of Tat-mediated transactivation. Overall, these findings indicate that the HTLV-2 APH-2 may affect the HIV-1 replication at multiple levels by (a) inhibiting the Tat-mediated transactivation and (b) hampering the virus release by affecting the cellular gag expression.

## 1. Introduction

Human T-cell Leukemia Virus Type 1 (HTLV-1) is a retrovirus associated with myelopathy/tropical spastic paraparesis, leukemia/lymphoma, impaired immune responses to *Strongyloides stercoralis,* bronchiectasis, and inflammatory disorders such as uveitis, arthropathy, infectious dermatitis, exocrinopathy, myositis, and others [1]. HTLV-2 is less pathogenic and found to be associated with lymphocytosis and certain inflammatory conditions [2,3]. HTLV-1 and HTLV-2 encode the negative regulatory proteins HTLV-1 bZIP protein (HBZ) and APH-2, respectively [4,5]. These 3′ transcribed proteins regulate Tax (HTLV Trans activating protein) mediated transcription. HTLV-2 anti-sense protein (APH-2) is known for its persistent expression and regulation of transcriptional processes by engaging cellular proteins CREB, CBP, NF-kB, and viral protein Tax-2 [5,6,7,8,9,10,11]. APH-2 can also modulate cellular and viral pathways by affecting transforming growth factor (TGF-β) and interferon regulatory factor (IRF-1) signaling [12].

Due to common modes of transmission, in case of an established HIV/HTLV co-infection, viral proteins may interact with each other and with host proteins. These protein interactions may have an effect on the replication of both coinfecting pathogens, altering the course and severity of the disease. Prior studies have reported beneficial or at times detrimental effects of HTLV co-infection on the HIV-1 disease progression [13,14,15,16,17,18]. As both are retroviruses, they share considerable genetic homology. The regulatory proteins Tax and Rex of HTLV can recognize and bind to the respective cognate sites of HIV-1 Tat and Rev, resulting in the functional replacement for HIV-1 regulatory proteins [19]. Studies have also reported that HTLV Tax protein alters chemokine expression and impacts HIV-1 disease progression [13,16,18,20,21,22,23]. Similarly, the CREB transcription factor, which is effectively important in HTLV transcription, was also found to play a key role in the HIV-1 Tat-mediated transcription [24,25,26]. In addition, NF-kB, a key host transcription factor, utilized by HIV-1 for transcription, has also been found to interact with APH-2 [10,11,12].

Although the earlier studies were mostly focused on the HBZ, a counterpart of APH-2 in HTLV-1, which was shown to reduce Tax-mediated transcription of HTLV-1 replication, Gaudray et al., while studying the effect of HBZ on HTLV-1 transcription, assessed the role of HBZ on HIV-1 Tat- and LTR-mediated transcription and indicated elevated levels of Tat-driven LTR transcription by HBZ [4]. This could be attributed to the functional differences between HBZ and APH-2 [12], leading to contradictory effects on HIV-1 transcription. A recent study by Martini et al. highlighted the association of APH-2 with a component of the host endosomal-sorting complex required for transport (ESCRT) [27]. ESCRT-0 hepatocyte growth factor regulated tyrosine kinase substrate (HRS) recruits ESCRT-I by interacting with TSG 101, followed by recruitment of complex II and III sequentially to promote budding and release of vesicles. Several viruses, including HIV-1 for efficient viral particle release, hijack this ESCRT machinery of the host. HIV-1 Gag p6 mimics its PSAP sequence motif with HRS for binding and recruitment of TSG 101 for ESCRT-mediated efficient viral particle budding and release [28,29,30]. Altogether, the interaction of APH-2 with HRS and HRS PSAP mimicking the strategy of HIV-1 Gag for ESCRT-mediated HIV-1 budding and release suggest that there could be a possibility of HIV-1 Gag PSAP and APH-2 interactions, leading to an altered course of HIV-1 release. Considering these aspects, understanding the role of APH-2 in HIV-1 replication is crucial.

As the common players are affecting various replication steps of these two viruses, there might be some mutual intersecting factors impacting each other. We, therefore, hypothesized that APH-2 could interfere with HIV-1 replication and influence different stages of the HIV-1 lifecycle. Here, we report that the APH-2 reduces HIV-1 virus production and/or virus release, accompanied by the ability of APH-2 to impede the HIV-1 Tat-mediated transcription. We also identified that the N-terminal IXXLL motif of APH-2 is an essential determinant required for depletion of Tat-mediated transcription.

## 2. Materials and Methods

### 2.1. Cell Lines and Plasmids

In this study, 293T (ATCC-CRL-3216) and TZM-bl (RRID: CVCL_B478) cell lines, obtained from the NIH AIDS reference reagent program (NIH-ARRP), USA. maintained in Dulbecco’s Modified Eagle’s medium, supplemented with 10% fetal bovine serum (FBS), penicillin (100 units/mL), and streptomycin (100g/mL), were used.

Plasmids HIV-1 pNL4.3, HXB2 pGag-EGFP, pcDNA3.1+/Tat101-flag (PEV280), and pBlue3’LTR-luc-B were obtained from the NIH-ARRP. The HTLV-2 APH-2 was amplified from the HTLV-2 molecular clone pH6neo (gifted by Dr.P.G. Green, USA) and cloned into the pCMV-HA expression vector to generate the HA-APH-2. The mutant versions HA-APH-2_LXXLL amino acid (aa) 64–68, changed to aa AXXAA, and HA-APH-2_IXXLL aa 179–183 to aa AXXAA, were generated by site-directed mutagenesis.

### 2.2. Virus Release and Infectivity Assays

To assess the role of APH-2 on HIV-1 replication, 293T cells (ATCC-CRL-3216) were seeded at the density of 5 × 10^5^ cells per well of a 6-well plate. The cells were co-transfected with HA-APH-2 and HIV-1 pNL4.3 on the next day using a calcium phosphate transfection kit (Promega) following the manufacturer’s instructions. After 48 h, the culture supernatant was cleared by centrifugation and passed through a 0.45 μm syringe filter. A part of the supernatant was used for the infectivity assay in TZM-bl cells.

The centrifuged and filtered virus supernatant was pelleted by layering the clear culture supernatant (1 mL) on 250 µL of 20% sucrose cushion followed by centrifugation at 25,000× *g* for 90 min at 4 °C. The pelleted virions were dissolved in 60 µL of 2 × SDS sample-reducing buffer. The cell lysates were prepared by washing cells with cold PBS on ice and treatment with cell lysis buffer (50 mM Tris HCl (pH 7.5), 120 mM NaCl, 1% Triton X-100 and protease inhibitor cocktail (SIGMA)). The pelleted virions and cell lysates were analyzed by SDS-PAGE and Western blot using anti-HIV-1 p24 monoclonal antibody (Clone 183-H12-5C, NIH-ARRP). The expression of HA-APH-2 was identified using monoclonal anti-HA-peroxidase antibody (SIGMA) at a dilution of 1:6000 of anti-mouse IgG HRP 1:3000 (Thermo Fisher Scientific). Simultaneously, released virions were titrated on the TZM-bl cells. Equal volumes of cleared supernatants (50 µL) were used to infect the TZM-bl cells and the luciferase activity was measured using the britelite plus luciferase assay kit (PerkinElmer) after 48 h.

### 2.3. APH-2 and Virus-Like Particle Release

HXB2 pGag-EGF, characterized by Rev-independent expression of Gag-EGF, and production of virus-like particles with an efficiency equivalent to that of the HIV-1 Gag in full-length virus, was co-transfected along with varying concentrations of CMV HA-APH-2. The culture supernatant was cleared by centrifugation and passed through a 0.45 µm syringe filter, 24 h post-transfection. Virus supernatant was then pelleted by using 20% sucrose gradient centrifugation. Subsequently, cell lysates were prepared using cell lysis buffer (50 mM Tris HCl (pH 7.5), 120 mM NaCl, 1% Triton X-100 and protease inhibitor cocktail (SIGMA)). Cell lysate and virus-like particle supernatant was then subjected to the Western blot analysis with anti-HA peroxide (sigma) and anti-HIV-1 p24 monoclonal antibody (Clone 183-H12-5C, NIH-ARRP).

### 2.4. Cellular Gag mRNA Quantification

The total RNA from the cell lysate was extracted using the Qiagen total RNA extraction kit (Qiagen), and the extracted RNA was used to synthesize cDNA with the help of the superscript IV cDNA synthesis kit (Invitrogen). Random hexamers were used for cDNA synthesis. A relative quantification assay was performed to detect the cellular expression of HIV-1 gag mRNA using gag-specific primers and SYBR select master mix. For relative quantification of the gag gene, the ΔCt (cycle threshold) value of each sample was calculated by subtracting the Ct value of endogenous control (β-actin) from the target (gag). Furthermore, the ΔΔCt value was measured by deducting the average ΔCt value of the experimental control (pNL4.3 transfected cells’ RNA) from the samples (pNL4.3 plus HA-APH-2 transfected cells’ RNA). This ΔΔCt value was used to measure the percentage of gag expression for each sample.

### 2.5. Virus Release Efficiency

The efficiency of the virus release was calculated as the amount of virion-associated p24 as a fraction of the total amount of Gag (virion-associated p24, plus all the cell-associated expressed precursors of Gag). The protein band intensities were measured using NIH Image J software.

### 2.6. Assays for Detection of Role of APH-2 on HIV-1 Transcription

To identify the ability of APH-2 to affect the Tat-mediated transactivation of transcription, assays were performed in the 293T cell line by co-transfecting the pCMV-HA-APH-2- and LTR-based luciferase reporter gene constructs (pBlue3’LTR-luc) in the presence of Tat expression vector (pCDNA3.1 + Tat-Flag) in the 293T cell line. The cells were seeded in 24- or 6-well plates at a density of 4 × 10^4^ or 5 × 10^5^ cells per well, respectively. Cells were transfected after 24 h of incubation with the lipofectamine 2000 reagent (Invitrogen) according to the manufacturer’s instructions. At 24 h post-transfection, luciferase activity was measured using the britelite plus luciferase assay kit (PerkinElmer).

### 2.7. Immunoprecipitation

pCMV-HA-APH-2 and pCDNA3.1 + Tat-Flag protein interaction was studied by immunoprecipitation. Briefly, the protein of interest, together or separately, was transfected in 293T cells, using lipofectamine 2000 (Invitrogen). Twenty-four hours post-transfection, the cells were harvested, washed with ice-cold PBS and lysed with Triton-X supplemented with protease cocktail. The cell lysates were mixed with anti-Flag antibody (Abcam) and the conjugated protein G beads (SIGMA) were incubated at 4 °C overnight with continuous shaking. Beads were washed three times with IP buffer, boiled in 2X SDS-PAGE loading buffer for 5 min and co-immunoprecipitations were analyzed by Western blot.

## 3. Results

### 3.1. Dose-Dependent Effect of HTLV-2 APH-2 Expression on HIV-1 Release and Infectivity

**Figure 1:**
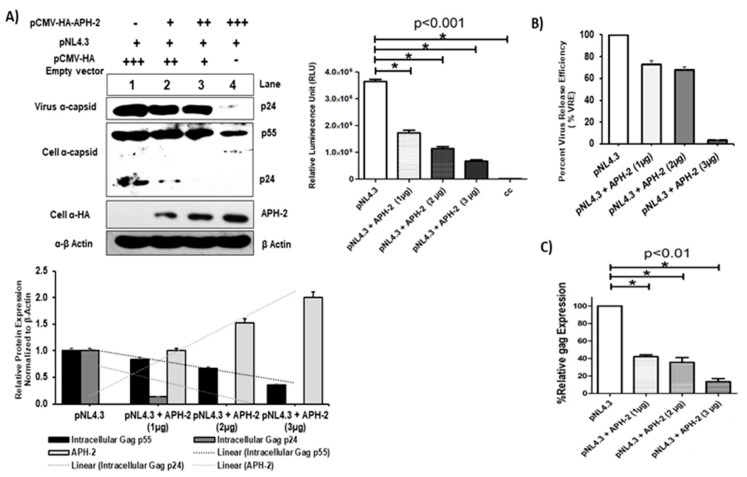
APH-2 overexpression reduces HIV-1 Gag production and viral release. (**A**) Effect of APH-2 on full-length HIV-1 pNl4.3: 293T cells were transfected with 1 µg pNL43 HIV-1 full-length clone only, as well as with pNL4.3 and increasing amounts (1–3 µg) of plasmid-encoding HA-APH-2. Western blot was performed to examine cell-free virus production (capsid protein, p24) (Panel 1), cellular Gag precursors (Panel 2) and APH-2 expression (Panel 3). Cellular beta actin expression represents the loading control (panel 4). (Amount of plasmid used is represented in figure as, − = 0 μg; + = μg; ++ = 2 μg; +++ = 3 μg). Graph below shows densitometric analysis of Western blot performed using Image J software, and relative cellular protein expression normalized to β-actin. Graph in the upper right part of the figure shows the infectivity of released virions in the TZM-bl single-cycle infectivity assay (*n* = 3 ± standard deviation). *p*-values were determined using a Student’s *t* test. * *p* < 0.001. (**B**) HIV-1 virus release efficiency: Densitometric analysis was performed using NIH Image J software and graph was plotted for average percent change in Gag expression. The efficiency of virus release was calculated as the amount of virion-associated p24, as a fraction of the total amount of Gag (virion-associated p24 plus all the cellular precursors of Gag) relative to pNL4.3, which was arbitrarily set at 100. (**C**) Relative gag expression in the presence of APH-2: Cell lysate on post-transfection subjected to total RNA isolation followed by real-time qPCR for measurement of relative gag mRNA expression, gag expression normalized with endogens control B-actin and percent gag expressed with and without APH-2 are shown in the graph. Cellular gag expression in 293T cells (*n* = 3 ± standard deviation), *p*-values were determined using a Student’s *t* test. * *p* < 0.01.

To investigate the key role of APH-2 on HIV- 1 replication, pNL4.3 and HA-APH-2 were co-transfected in 293T cells with increasing concentrations of HA-APH-2. The titer of the released virions was carefully assessed in TZM-bl cells. A significant reduction of HIV-1 p24 expression was observed in the infected culture supernatants by immunoblot analysis (Figure 1A, upper left, panel 1). The expression of cellular Gag precursors (p55 and p24) was also downregulated (~20%) in the presence of APH-2 (Figure 1A, upper left, panels 2 and 3, lanes 1 to 4, and Figure 1A, lower part, graph). The released virus infectivity (or virus production) data showed a decline in HIV-1 p24 expression (panel 1) as well as a dose-dependent decrease in luciferase expression in the presence of APH-2. A maximum decrease of up to 75–80% in the luciferase activity was observed at the highest concentration of APH-2 (Figure 1A, upper right), indicating a significant reduction of viral infectivity in the presence of APH-2. The estimated virus release efficiency data also supported the finding and indicated a drastic change in viral release efficiency in the presence of APH-2 (Figure 1B).

**Figure 2:**
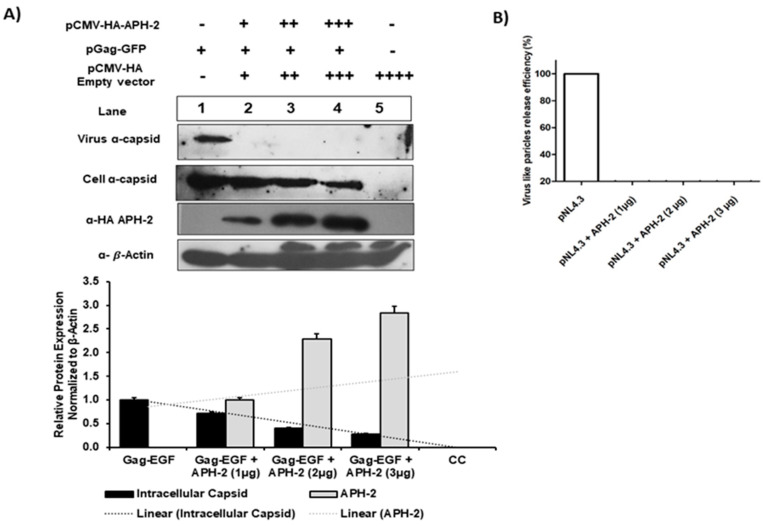
Role of APH-2 in HIV-1 release. (**A**) Effect of APH-2 on pGag-EGFP: 293T cells were transfected with HXB2 pGag-EGF (1 µg) only and co-transfected with HXB2 pGag-EGF (1 µg), along with increasing amounts of plasmid-encoding HA-APH-2 (1–3 µg). Western blots were performed to examine cell-free virus-like particle production (Panel 1), cellular Gag (pr55-EGFP) expression (Panel 2) and APH-2 expression (Panel 3). Bottom panel: Cellular beta actin expression represents the loading control. (Amount of plasmid used is represented in figure as − = 0 μg; + = μg; ++ = 2 μg; +++ = 3 μg; ++++ = 4 µg). The graph below shows densitometric analysis of Western blot performed using Image J software, and relative cellular protein expression normalized to β-actin. (**B**) HIV-1 virus release efficiency: Densitometric analysis was performed using NIH Image J software and a graph was plotted for average percent change in Gag expression. The efficiency of virus release was calculated as the amount of virion-associated mature capsid protein, p24, as a fraction of the total amount of Gag (virion-associated p24 plus all the cellular precursors of Gag) relative to pGag-EGFP, which was arbitrarily set at 100.

As APH-2 affected the virus release from 293T cells in the case of an infectious replication-competent pNL4.3 molecular clone, we further investigated whether a similar effect can be observed with the HXB2 pGag-EGFP expression vector. The co-transfection of pGag-EGFP and HA-APH-2 in 293T cells with increasing concentrations of APH-2 and protein expression analysis showed that the virus release was severely affected even at the lowest concentrations of APH-2 (1 µg) (Figure 2, upper part, panel 1). Similarly, there was a dose-dependent decrease in the internal Gag production in the presence of APH-2 (Figure 2, upper part, panel 2). This finding was also supported by the densitometric analysis of the western blot (Figure 2, lower part, graph).

### 3.2. Role of APH-2 in HIV-1 Transcription

Experimental results showed that HIV-1 release was significantly affected in the presence of APH-2. In addition, the real-time PCR experiments carried out to quantitate mRNA expression showed that the expression of Gag at the mRNA level ranged between 10% and 40% at 1–3 µg/mL and was found to be dose-dependent (Figure 1C). The result further suggests the possible role of APH-2 in the early stages of HIV-1 replication.

**Figure 3:**
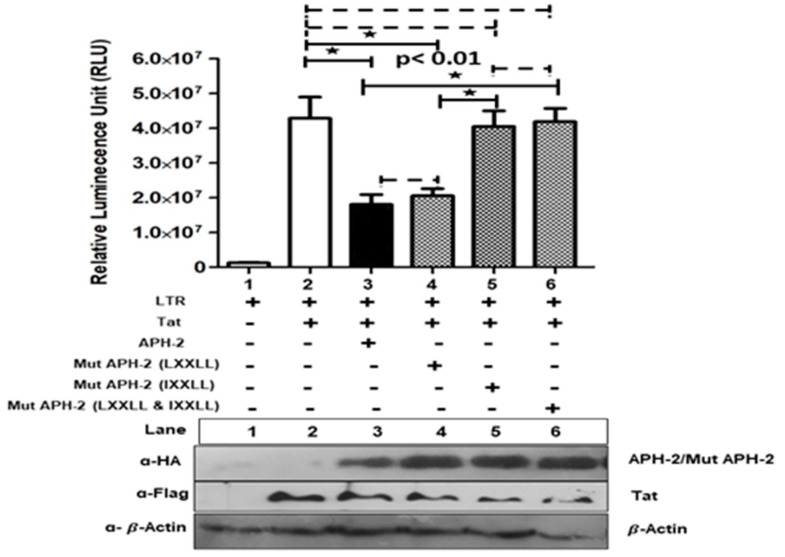
Effect of APH-2 on HIV-1 Tat-mediated transcription: 293T cells were transfected with pBlue3′LTR (1 µg) only and co-transfected with pCDNA3.1 Tat 101-flag (3 µg), HA-APH-2 (3 µg), as well as mutant versions of HA-APH-2 (3 µg), (+ and − sign used to represent presence and absence of plasmid used for transfection) as shown in the figure. The graph represents the luciferase activity measured 24 h post-transfection (*n* = 3 ± SD). *p*-values were determined using a Student’s *t* test. * *p* < 0.01. Dashed lines indicate no significant difference. The bottom panel represents the cellular expression of pCDNA3.1 Tat 101-flag, HA-APH2 and HA-APH-2 mutants. Cellular beta actin expression represents the loading control.

To address the role of APH-2 on Tat-mediated HIV-1 transcription, HA-APH-2/Mut APH-2 was co-transfected with pcDNA3.1+/Tat101-flag and HIV-1 LTR-Luc with different combinations. Overexpression of APH-2 demonstrated the hampered ability of HIV-1 Tat to transactivate expression of luciferase from HIV-1 promoter, resulting in a reduction of Tat-mediated transcription (up to 66%). Further, mutational analysis (Supplementary Figure S1, schematic representation for APH-2 mutant generation) to identify the APH-2 domains responsible for transcriptional hindrance showed that the mutant containing an altered IXXLL motif at the N-terminal domain of APH-2 was responsible for the reverted inhibitory effect imposed by APH-2. The mutants of previously reported CREB-binding LXXLL motifs of APH-2 did not fully restore the luciferase activity (Figure 3, graph, column 5), but the mutant APH-2 IXXLL restored the activity almost equal to the luciferase expression of the Tat control (Figure 3, graph, column 5). Similarly, APH-2 containing mutations at both the domains (Mut HA APH-2, LXXLL and IXXLL) also showed fully reverted luciferase activity. These results indicate that the IXXLL motif might play an important role in the reduction of Tat-mediated transcription by APH-2. The expression of Tat and APH-2 protein in the Western blot is shown in Figure 3.

### 3.3. Association of Tat and APH-2

**Figure 4:**
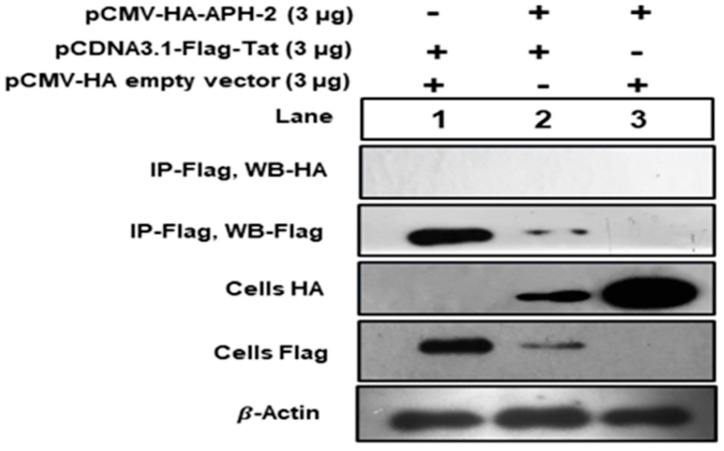
Immunoprecipitation of APH-2 and HIV-1 Tat association: 293T cells were co-transfected with HA-APH-2 (3 µg) and pCDNA3.1 Tat-Flag (3 µg), together and separately. Cell lysates were immunoprecipitated with anti-flag antibody, followed by Western blot with anti-HA. Cellular beta actin expression represents the loading control (+ and − sign used to represent presence and absence of plasmid used for transfection).

To study the association between Tat and APH-2, 293T cells were co-transfected with HA-APH-2 and Flag-Tat, together and separately. Cell lysates were immunoprecipitated with anti-flag antibody, followed by Western blot with anti-HA. The results indicated that there was no observed band for APH-2 after immunoprecipitation (Figure 4, panel 1, lanes 2 and 3), while there were visible bands for APH-2 expression in the cell lysate counterpart on anti-HA (Figure 4, panel 3, lanes 2 and 3). The expression of Tat was observed in both IP anti-flag and the anti-flag cell lysate counterpart (Figure 4, panels 2 and 4, lanes 1 and 2), which clearly indicated that there was no specific association observed between these two proteins under our experimental condition.

## 4. Discussion

The negative regulator APH-2 crucially controls HTLV-2 replication by influencing transcription [6,7]. Recent studies also explored its role in managing viral release and budding [27]. Considering the common aspects between HIV-1 and HTLV-2 such as lineage, mode of transcription, replication and the clinical impact of co-infection over the disease progression of HIV-1, there might be a possible intervening effect of viral regulators on replication of one another. Our study clearly revealed that APH-2 has an ability to intervene in HIV-1 replication at several stages. Overexpression of HA-APH-2 severely affected the virus release, along with the intracellular Gag expression (Figure 1A,B), suggesting the plausible effect of HA-APH-2 on the late events of HIV-1 replication.

Our findings of the virus production/infectivity assay from the HIV-1 full-length clone, the estimated virus release efficiency and the amount of virus-like particles produced from HXB2 pGag-EGF in the presence of APH-2 cumulatively indicate that APH-2 is responsible for impaired HIV-1 release. In line with our findings, Martini et al. also showed a regulatory role of APH-2 in HTLV-2 viral release and budding. This novel role of the negative regulator APH-2 is mainly mediated by interactions with the host ESCRT machinery. Furthermore, findings have shown that the IXXLL motif has an interaction with HRS protein, a component of ESCRT-0. As HRS contains a proline-rich domain including the PSAP motif, the ability of APH-2 to interact with the PSAP motif is important for the negative regulation of HTLV-2 virus release. It is evident that HIV-1 imitates the PSAP domain of HRS, and was found to be located in the late domain of Gag at the P6 region. Suitable interaction of Gag P6-PSAP and TSG 101 (ESCRT-I) leads to efficient budding and release of HIV-1 virion [28,29,30]. Together, our study findings highlight the possibility of a Gag PSAP and APH-2 interaction responsible for impaired HIV-1 viral release in the presence of APH-2.

The present study identified that the overexpression of APH-2 has a significant effect on the relative gag transcripts’ expression. The findings directed us to identify the role of APH-2 in Tat-mediated transcription, which revealed inhibition of Tat-mediated transactivation of HIV-1 LTR in the presence of APH-2. Moreover, a mutation in the IXXLL motif at the N-terminal domain of APH-2 resulted in the reversion of the inhibitory effect imposed by APH-2. Our findings highlight the functional involvement of the IXXLL motif. These results highlighting the importance of the N-terminal region of APH-2 and are compatible with the study by Martini et al., which identified a similar domain of APH-2 for the interaction with the PSAP domain of HRS [27]. Although the Tat-mediated transcription was affected in the presence of APH-2, we did not find a specific association between APH-2 and Tat, indicating that HTLV-2 APH-2 may not directly engage HIV-1 Tat to impose its inhibitory effect. We also observed that there is a reduction in the expression of both proteins when they are transfected together, as compared to the protein expression when they are transfected individually. To the best of our knowledge, only one prior study by Torresilla et al. [23] investigated the role of APH-2 in HIV-1 replication. The authors reported increased MIP1α levels along with the inhibition of NF-kB and NFAT in the presence of APH-2, but observed positive modulation of full-length HIV-1 luciferase expression [23]. Even though the study reported increased expression of MIP1 α, downregulation of NF-kB and NFAT supposedly supports the negative role of APH-2 on HIV-1 replication. Furthermore, their experiments with full-length HIV-1 showed enhanced luciferase expression in the presence of APH-2, suggesting differing positive modulation of HIV-1 replication. In contrast to these results, we have observed decreased HIV-1 capsid expression and transcription in the presence of APH-2. Overall, our findings deviate, and support a negative role of APH-2 in HIV-1 replication. The effect of APH-2 over NF-kB and NFAR [10,11,31] aligns with the possibility of involvement of host transcriptional players and APH-2 to hinder the HIV-1 transcription. Earlier studies reporting an interaction of APH-2 with different transcription and co-transcription factors, such as CREB, NFKB, c-Jun, JunB, etc. [10,11,26,32,33], also support our study findings, and could be a possible reason for the negative role in the viral transcription.

A study carried out by Gaudray et al. [23] observed an effect of HTLV-1 HBZ protein on HIV-1 LTR transcription, while in the present study, the HTLV-2 counterpart APH-2 has shown exactly the opposite effect on HIV-1 transcription and was found to be hindering the HIV-1 transcription. Hence, our study hints towards a differential fate of the HIV-1 progression during HTLV co-infection and emphasizes on the important role played by the negative regulators of HTLV.

To conclude, APH-2 was found to have an impact on HIV-1 replication, cumulatively affecting replication stages of early transcription and delayed viral release. This study identified the novel role of APH-2 in HIV-1 replication and helped to bridge the gap between the known regulatory role of APH-2 in HTLV-2 and HIV-1. Further studies are required to focus on protein interactions (Gag and APH-2, along with the APH-2-mediated fate of HIV-1 proteins Tat and Gag) to identify detailed insights for the mechanisms involved in APH-2 affecting HIV-1 replication.

## Data Availability

Data is contained within the article or supplementary material.

## Supplementary Materials

The following are available online at https://www.mdpi.com/article/10.3390/v13081432/s1, Supplementary Figure S1. Schematic representation for APH-2 mutant generation.
