# Supplementary material for: HTLV-2 Encoded Antisense Protein APH-2 Suppresses HIV-1 Replication
Source: Viruses. 2021 Jul 23;13(8):1432. doi: 10.3390/v13081432 (PMC8402832; doi:10.3390/v13081432)
Supplement: Supplementary file 1 [file viruses-13-01432-s001.zip › viruses-1285112-SI.pdf]

Supplementary Figure S1 : Schematic representation for generation of Mutants of APH-2

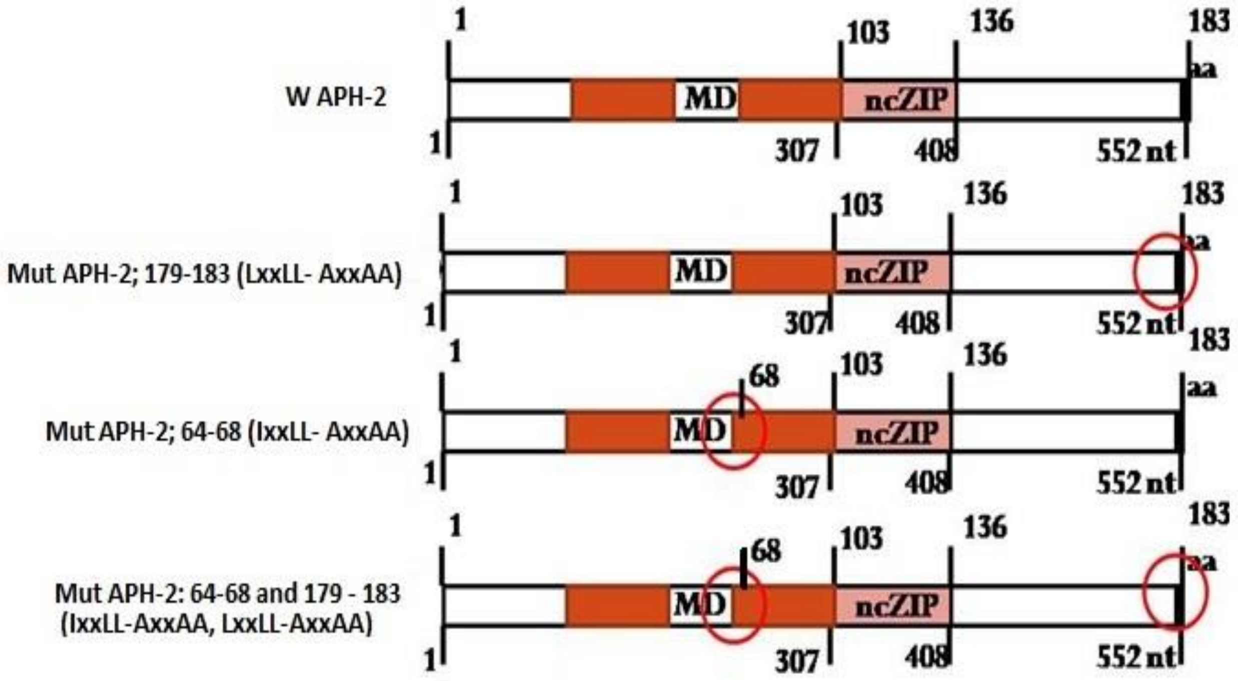

Supplementary Figure S1 : Schematic representation for generation of Mutants of APH-2, The HTLV-2 APH-2 was amplified from the HTLV-2 molecular clone pH6neo (gifted by Dr.P.G. Green, USA) and cloned in to the pCMV-HA expression vector to generate the wild type HA-APH-2. The mutant versions HA-APH-2_LXXLL, amino acid (aa) 64-68 changed to aa AXXAA and HA-APH-2_IXXLL aa 179-183 to aa AXXAA were generated by site directed mutagenesis.
